# CD8^+^ T-cell counts: an early predictor of risk and mortality in critically ill immunocompromised patients with invasive pulmonary aspergillosis

**DOI:** 10.1186/cc12836

**Published:** 2013-07-24

**Authors:** Na Cui, Hao Wang, Yun Long, Dawei Liu

**Affiliations:** 1 Department of Critical Care Medicine, Peking Union Medical College Hospital, Peking Union Medical College & Chinese Academy of Medical Sciences, Beijing, 100730, China

**Keywords:** CD8^+^ T-cell, Critically ill, Immunity, Immunocompromised patients, Invasive pulmonary aspergillosis

## Abstract

**Introduction:**

Critically ill immunocompromised (CIIC) patients with pulmonary infection are a population at high risk for invasive pulmonary aspergillosis (IPA). The host defenses are important factors to consider in determining the risk and outcome of infection. Quantification of changes in the status of host immunity could be valuable for clinical diagnosis and outcome prediction.

**Methods:**

We evaluated the quantitative changes in key humoral and cellular parameters in CIIC patients with pulmonary infection and their potential influence on the risk and prognosis of IPA. We monitored the evolution of these parameters in 150 CIIC patients with pulmonary infection on days 1, 3 and 10 (D1, D3 and D10) following ICU admission. The primary outcome was 28-day mortality. Follow-up included 60- and 90-day mortality.

**Results:**

Among the 150 CIIC patients included in this study, 62 (41.3%) had microbiological evidence of IPA. Compared with patients without IPA, CD3^+^, CD8^+^, CD28^+^CD4^+^ and CD28^+^CD8^+^ CD28^+^CD8^+^ T-cell counts (D1, D3 and D10) and B-cell counts (D1 and D3) were significantly reduced in patients with IPA (*P* < 0.05). Multivariate regression analysis revealed that CD8^+^ (D3 and D10) (odds ratio (OR) 0.34, 95% confidence interval (CI) 0.23 to 0.46; OR 0.68, 95% CI 0.56 to 0.80), CD28^+^CD8^+^ (D3) (OR 0.73, 95% CI 0.61 to 0.86) and CD3^+^ (D10) (OR 0.81, 95% CI 0.63 to 0.98) T-cell counts were independent predictors of IPA in CIIC patients. Receiver operating characteristic analysis of immune parameters predicting 28-day mortality revealed area under the curve values of 0.82 (95% CI 0.71 to 0.92), 0.94 (95% CI 0.87 to 0.99), and 0.94 (95% CI 0.85 to 0.99) for CD8^+^ T-cell counts (D1, D3 and D10, respectively) and 0.84 (95% CI 0.75 to 0.94), 0.92 (95% CI 0.85 to 0.99) and 0.90 (95% CI 0.79 to 0.99) for CD28^+^CD8^+^ T-cell counts (D1, D3 and D10, respectively). Kaplan-Meier survival analysis provided evidence that CD8^+^ and CD28^+^CD8^+^ T-cell counts (<149.5 cells/mm^3^ and <75 cells/mm^3^, respectively) were associated with early mortality in CIIC patients with IPA (logrank test; *P* < 0.001).

**Conclusions:**

CD8^+^ and CD28^+^CD8^+^ T-cell counts were significantly lower in CIIC patients with IPA than in non-IPA patients. Lower CD8^+^ and CD28^+^CD8^+^ T-cell counts in CIIC patients with pulmonary infection were associated with higher risk and early mortality in IPA and may be valuable for clinical diagnosis and outcome prediction.

## Introduction

The number of community and hospitalized patients with compromised host defenses has increased dramatically in recent years. This increase is due mainly to the significant progress in transplantation procedures, the rapid development of cancer chemotherapy and immunotherapy and the broader use of corticosteroids. A significant number of immunocompromised patients who develop serious complications related to treatment toxicity and immunological dysregulation need to be transferred to the ICU for advanced supportive care [1]. Pulmonary complications are the most common cause of ICU admission in critically ill immunocompromised (CIIC) patients, and these are associated with high mortality rates of 30% to 90% [2]. Severe infection has been estimated to be another major cause of ICU admission and usually presents with pulmonary lesions coupled with respiratory dysfunction and radiographic infiltrates. Furthermore, a variety of opportunistic infections, including invasive pulmonary aspergillosis (IPA), cytomegalovirus (CMV) and *Pneumocystis carinii* pneumonia (PCP) are increasingly being identified as the main causes of pulmonary infections in CIIC patients.

*Aspergillus* has become a leading cause of severe fungal infections in CIIC patients [3]. The prevalence of IPA has been rising and currently ranges between 2% and 26%. The lung is the primary site of *Aspergillus* infection, and IPA has a high mortality rate of 74% to 92% [4]. Early diagnosis and effective antifungal therapy are important in decreasing the mortality associated with IPA. However, the superimposition of the compromised host defenses and critical illness, including atypical clinical presentation, poor diagnostic yield of cultures and especially the difficulty in obtaining samples of infected tissues, make the detection and management of IPA in CIIC patients difficult. Late diagnosis and antifungal therapy are associated with severe morbidity and a high mortality rate.

*Aspergillus*-related diseases are associated with a spectrum of immune disorders. Impaired host immunity predisposes patients with these diseases to infection with some specific pathogens that are associated with immunocompromise [5]. Even certain easily measurable real-time indicators, such as absolute neutrophil count and T-lymphocyte count, could provide important information regarding risk level and probable pathogens in immunocompromised patients. Therefore, we conducted this prospective study in CIIC patients with pulmonary infection to assess the discriminatory ability of key immunological parameters in predicting risk and prognosis in cases of IPA.

## Materials and methods

### Study population

All critically ill patients (age 14 years or older) with appropriate immunocompromised host factors and respiratory dysfunction for suspected or documented pulmonary infection admitted to the general ICU of the Peking Union Medical College Hospital (PUMCH) between October 2009 and December 2011 were prospectively and consecutively included in this study. PUMCH is a 2,000-bed university hospital with more than 400 immunology, hematology and oncology beds. The study was approved by the Ethics Committee of PUMCH, and written informed consent was obtained from the patients or their families.

### Inclusion criteria

Patients included in the study were age 14 years of age or older, had appropriate immunocompromised host factors and required intensive care for respiratory dysfunction combined with suspected or documented pulmonary infection. According to the 2008 European Organization of the Research and Treatment of Cancer/Mycoses Study Group (EORTC-MSG) consensus definitions [6], immunocompromised host factors included (1) receipt of a solid organ or hematopoietic stem cell transplant; (2) hereditary immunodeficiency, tumor, hematological malignancy and connective tissue disorders; and (3) receipt of immunosuppressive agents, for example, corticosteroids or T-cell immunosuppressants such as calcineurin inhibitors, anti–tumor necrosis factor α agents, antilymphocyte antibodies or purine analogues.

Diagnosis of pulmonary infection was defined as the presence of a new or progressive radiographic infiltrate plus at least two of the following [7]: (1) cough, purulent sputum or auscultatory findings of pulmonary consolidation; (2) dyspnea, tachypnea or hypoxemia; and (3) identification of pathogens isolated from cultures of the respiratory tract, sputum or blood samples. In addition, patients with pulmonary infection had to have two or more of the following clinical features [8]: (1) body temperature 38°C or higher or less than 36°C, (2) respiratory rate 30 or more breaths/min, (3) pulse rate 120 or more beats/min and (4) abnormal total peripheral white blood cell counts greater than 10,000/mm^3^ or less than 4,000/mm^3^ or immature neutrophils less than 15%.

IPA was diagnosed on the basis of clinical, microbiological, radiological and histopathological criteria. Two independent senior radiologists who were blinded to clinical diagnosis reviewed all high-resolution computed tomography (CT) scans in shifts. On the basis of the 2008 EORTC-MSG consensus definitions, the presence of one of the following three signs on CT scans was considered to be suggestive of invasive aspergillosis: a well-circumscribed dense lesion (with or without a halo sign), an air crescent sign and a cavity. Because of the inherent uncertainty of the EORTC-MSG “probable” and “possible” classification terms in capturing true-positive IPA, in this study proven and probable IPA were considered to be true-positive IPA and possible IPA and no IPA were defined as true-negative cases.

Regarding the culture-independent serum antigen detection tests, the (1,3)-β-d-glucan (BG) assay was not included in the routine confirmation of IPA diagnosis, because a previous validation study in our unit indicated an unexpectedly low specificity. The galactomannan (GM) index also was not included in the diagnostic workup for unapproved conditions. Owing to the strong influence of traditional cultures and potentially devastating complications of autopsy and biopsy, the latter are still not widely accepted by most Chinese people.

### Exclusion criteria

Exclusion criteria included one or more of the following: pregnancy or lactation, lack of proper immunocompromised host factors and insufficient evidence of pulmonary infection.

### Clinical and laboratory evaluation

#### Clinical assessments

All patients underwent a comprehensive clinical assessment at the time of ICU admission, including age, sex and underlying diseases associated with the development of IPA in critically ill patients [9], antifungal treatment at least 48 hours previously and important biological parameters (levels of creatinine, albumin and blood glucose) at ICU admission. Immunocompromised host factors were divided into “underlying immunocompromised disease” and “receipt of immunosuppressive agents.” Acute Physiology and Chronic Health Evaluation II (APACHE II) [10] and Sequential Organ Failure Assessment (SOFA) [11] scores were computed on the first day following ICU admission. Organ failure was defined as a SOFA score of 3 or higher [12]. Life-sustaining treatments (need for mechanical ventilation (MV), vasopressors or renal replacement therapy) for 24 hours or longer were recorded based on clinical evaluation and recent recommendations [13]. Treatment-related factors, including the proportion of indwelling catheters, antibiotic treatment, steroid treatment, use of intravenous immunoglobulin and other clinically indicated investigations (for example, CT scan) were analyzed. Outcome analyses included survival evaluation, which was defined as the percentage of patients discharged alive from the ICU and from the hospital. Follow-up included 28-, 60- and 90-day mortality rates and was censored on 30 March 2012.

#### Microbiological diagnostics

All infections in this study were microbiologically documented and defined as fever with positive microbiological tests from a focus on infection and/or blood culture [14], and empirical antibacterial treatment was promptly initiated according to international recommendations [15]. Modifications of antibacterial therapy and addition of antifungal therapy were based on clinical course (persistent fever for longer than 72 hours and site of infection) and the results of the diagnostic procedures.

Chest CT images were demanded within 3 days of enrollment. Lower respiratory tract specimens were obtained three times weekly following ICU admission for at least 2 weeks. Because of the difficulty of sputum collection from patients with severe respiratory distress and progressive hypoxemia outside the ICU, almost all of the specimens of CIIC patients included in this study were collected in the ICU. Culture procedures were performed immediately after patient transfer to the ICU, and specimens were collected and sent to the PUMCH clinical microbiology laboratory within 2 to 4 hours. According to the Standard Sample Dispatching Procedure of the Department of Clinical Microbiology Laboratory in PUMCH, standard culture of respiratory secretion was defined as containing more than 25 neutrophils and less than 10 squamous epithelial cells per low-power field (×100 magnification). Quantitative culture by a quantitative or semiquantitative method is used to distinguish colonization from respiratory infection. Identification and sensitivity data were usually available within 48 to 72 hours. Gram staining, including the presence of polymorphonuclear leukocytes (PMNLs), macrophages and morphology of any bacteria, may improve diagnostic accuracy when correlated with culture results. The respiratory specimens were also screened for PCP, CMV and tuberculosis. The initial diagnostic workup included blood and urine cultures to confirm coinfection foci [16]. Serological studies were requested to rule out other atypical pneumonia pathogens (for example, anti-*Mycoplasma* and anti-*Legionella* antibodies). The follow-up included daily physical examinations and any other clinically indicated investigations (for example, CT scans). The results of the relevant lower respiratory tract cultures, CT scans and other etiological tests within the week prior to ICU admission were also considered in making the diagnosis of IPA.

Tracheobronchial aspirates (TBAs) were commonly used for the diagnosis of pulmonary infection in our hospital because of the limited availability of bronchoalveolar lavage (BAL)/protected specimen brush (PSB). TBAs have been defined as a reliable diagnostic strategy in the 2005 American Thoracic Society (ATS) guidelines on hospital-acquired pneumonia (HAP), the 2008 EORTC/MSG criteria on IPA and the 2009 European perspective on HAP [17]. The accuracy of TBAs for obtaining quantitative cultures of lower respiratory tract samples is comparable to that of bronchoscopic techniques [18]. Therefore, both noninvasive TBA and invasive diagnostic strategies such as BAL, PSB or its modifications could be used to provide lower respiratory tract specimens for diagnosis of HAP.

Patients were evaluated routinely at the time of ICU admission and during the first 3 days for acute noninfectious conditions that might be related to the respiratory dysfunction that required intensive care, based on clinical presentation. If a patient had respiratory dysfunction caused by noninfectious conditions, the patient was excluded from the study.

### Immunological laboratory workup

Blood was collected from each CIIC patient with IPA on days 1, 3 and 10 (D1, D3 and D10) following ICU admission. Peripheral blood mononuclear cells (PBMCs) were separated and stained with combinations of different fluorescent monoclonal antibodies followed by flow cytometric analysis (three-color EPICS-XL flow cytometer; Beckman Coulter, Brea, CA, USA) to detect T cells (CD3^+^), CD4^+^ T-cell subgroups (CD4^+^CD3^+^, CD28^+^CD4^+^), CD8^+^ T-cell subgroups (CD8^+^CD3^+^, CD28^+^CD8^+^), B cells (CD19^+^) and natural killer (NK) cells (CD3^–^CD16^+^CD56^+^). Serum levels of complement factor 3 (C3), complement factor 4 (C4), immunoglobulin A (IgA), IgG and IgM were measured by rate nephelometry (Array 360; Beckman Coulter).

The above key data were obtained and verified by PUMCH laboratories. Monitoring was conducted by an independent clinical research organization according to good clinical practice (GCP) and standard operating procedures in compliance with Chinese government regulations [19].

### Statistical analysis

Quantitative variables with normal distribution were expressed as the mean ± standard deviation (SD), and quantitative variables with non-normal distribution were expressed as the median and interquartile range (IQR). Categorical variables were compared using the χ^2^ test or Fisher’s exact test as appropriate. Continuous variables were compared using Student’s *t*-test or the Mann–Whitney *U* test. *P* values associated with “equal variances not assumed” were reported for variables that violated the homogeneity of variance assumption. Univariate and multivariate logistic regression analyses were performed to identify the immune parameters for prediction of IPA in CIIC patients, and the results were expressed as Wald index, *P* value and odds ratio (OR) with 95% confidence intervals (CIs). Variables that showed *P* < 0.05 in univariate analysis were included in the multivariate regression analysis model, and principal component analysis (PCA) was applied to adjust for the possible multicollinearity among independent variables (immune parameters measured on D1, D3 and D10).

The discriminatory ability of immune parameters for predicting 28-day mortality in CIIC patients with IPA were determined by ROC curve analysis. The reliabilities and consistencies of diagnostic tests were assessed by calculating their sensitivity, specificity, positive predictive value and negative predictive value. Kaplan-Meier survival analysis was used to construct survival curves, and comparisons of survival distributions were based on the logrank test. Data processing was performed using SPSS version 13.0 software (SPSS, Chicago, IL, USA). All statistical tests were two-sided, and *P* < 0.05 was considered significant.

## Results

### Patients’ characteristics

**Figure 1:**
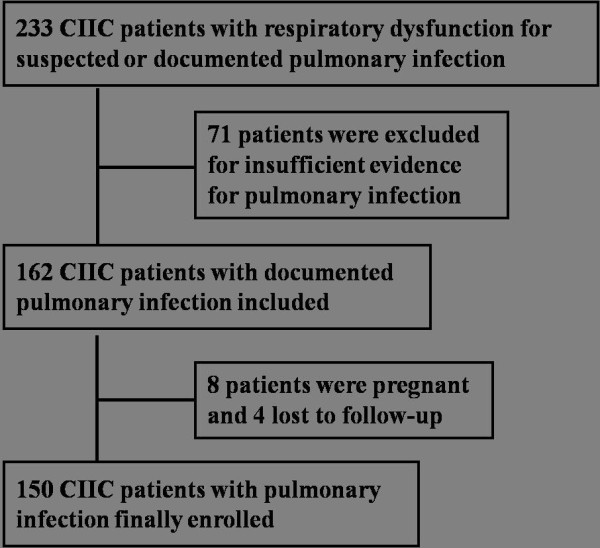
Diagram of study flow.

As reported in Figure 1, 233 CIIC patients with respiratory dysfunction caused by suspected or documented pulmonary infection were admitted to the ICU during the study period, including 71 who were excluded for noninfectious conditions. Among the remaining 162 CIIC patients, 8 were pregnant and 4 were lost to follow-up. In the end, 150 patients formed the basis of this study.

The main characteristics of study patients at ICU admission are shown in Table 1. On the basis of clinical, radiological, and microbiological evidence as defined by the 2008 EORTC-MSG consensus definitions, probable IPA was diagnosed in 62 patients (IPA group). No patient with proven IPA was diagnosed because of the preclusion of tissue biopsy and/or autopsy. Of the 150 CIIC patients, resource, hospital length of stay (HLOS) before ICU admission, and frequency of underlying diseases such as chronic obstructive pulmonary disease (COPD), diabetes mellitus, liver cirrhosis/hepatic failure, and chronic renal failure, which have been described in association with development of IPA in critically ill patients, did not differ between the IPA and non-IPA groups. Compared with the non-IPA group, no difference was found in most of the host factors except the proportions of patients diagnosed with microscopic polyangitis (MPA) (16.1% vs. 5.7%, p = 0.035) and the median total dose of steroid treatment [600.0 (720.0) mg vs. 480.0 (1000.0) mg, p = 0.023] being higher in the IPA group. We found no significant differences in the biological parameters, APACHE II score, and SOFA score at ICU admission between the patients with and without IPA.

**Table 1:** **Characteristics of patients at ICU admission**^**a**^

**Variables**	**All patients (*n*** **= 150)**	**No IPA (*n*** **= 88, 58.7%)**	**IPA (*n*** **= 62, 41.3%)**	**No IPA vs IPA *P *** **value**
Mean age (yr)	45.9 ± 19.8	42.3 ± 20.4	50.9 ± 17.9	0.009
Gender (males:females)	59:91	31:57	28:34	0.239
Resource (Emergency:Ward)	82:68	50:38	32:30	0.618
Mean HLOS before ICU admission (days)	9.0 (23.0)	9.0 (18.0)	18.9 ± 21.4	0.738
Underlying disease,^b^*n* (%)				
COPD	27 (18.0)	17 (19.3)	10 (16.1)	0.615
Diabetic mellitus	22 (14.7)	9 (10.2)	13 (21.0)	0.069
Liver cirrhosis/hepatic failure	17 (11.3)	7 (8.0)	10 (16.1)	0.12
Chronic renal failure	21 (14.0)	12 (13.6)	9 (14.5)	0.923
Immunocompromised host factors,^c^*n* (%)				
Immune system disease, *n* (%)	112 (74.7)	65 (73.9)	47 (75.8)	0.85
SLE	54 (36.0)	35 (39.8)	19 (30.6)	0.251
MCTD	19 (12.7)	12 (13.6)	7 (11.3)	0.671
MPA	15 (10.0)	5 (5.7)	10 (16.1)	0.035
Others	24 (16.0)	13 (14.8)	11 (17.7)	0.625
Hematologic malignancy, *n* (%)	26 (17.3)	16 (18.2)	10 (16.1)	0.744
Solid tumor, *n* (%)	9 (6.0)	6 (6.8)	3 (4.8)	0.615
Transplantation,^d^*n* (%)	9 (6.0)	5 (5.7)	4 (6.5)	0.845
Steroid treatment,^e^*n* (%)	48 (32.0)	27 (30.7)	21 (33.9)	0.724
Steroid^e^ and T-cell immunosuppressant, *n* (%)	53 (35.3)	27 (30.7)	26 (41.9)	0.169
Steroid dosage^f^ (mg)	480.0 (1,250.0)	480.0 (1,000.0)	600.0 (720.0)	0.023
Chemotherapy, *n* (%)	27 (18.0)	20 (22.7)	7 (11.3)	0.086
Antifungal treatment,^g^*n* (%)	18 (12.0)	10 (11.4)	8 (12.9)	0.803
Biochemical parameters at ICU admission				
Creatinine level (μmol/L)	137.2 (97.3)	135.4 (100.0)	139.8 (94.5)	0.564
Albumin level (g/L)	28.6 ± 5.0	29.1 ± 5.3	27.9 ± 4.5	0.173
Blood glucose level (mg/dl)	171.1 ± 66.2	168.8 ± 68.6	174.5 ± 63.0	0.615
APACHE II score	18.3 ± 5.4	18.2 ± 5.4	18.4 ± 5.4	0.793
SOFA score	8.6 ± 3.9	8.3 ± 3.6	9.2 ± 4.2	0.165

^a^*APACHE II* Acute Physiology and Chronic Health Evaluation II, *COPD* chronic obstructive pulmonary disease, *HLOS* hospital length of stay, *MCTD* mixed connective tissue disease, *MPA* microscopic polyangitis, *IPA* invasive pulmonary aspergillosis, *SLE* systemic lupus erythematosus, *SOFA* Sequential Organ Failure Assessment. Continuous variables are expressed as the means ± SD or medians (IQR). All other data are raw numbers (%). ^b^This variable was defined as the predisposing conditions that have been described associated with the development of IPA in critically ill patients; organ failure was defined as a SOFA score of ≥3 for any system. ^c^Host factors were defined according to the IFD classification in the 2008 EORTC-MSG criteria [6]. ^d^Variable included solid-organ and hematopoietic stem cell transplantation. ^e^All steroid treatments indicated were defined according to the 2008 EORTC-MSG criteria: mean minimum dose of 0.3 mg/kg/day prednisone equivalent for more than 3 weeks before ICU admission. Other corticosteroids should be converted into equivalent dose of prednisone before calculation and comparison. ^f^Variable defined as total amount of steroid treatment, alone and combined with other immunosuppressive agents. ^g^Variable defined as treatment with systemic antifungal drugs including fluconazole, voriconazole, caspofungin and amphotericin B for 48 hours or more in the preceding week.

The clinical characteristics of the 150 CIIC patients are presented in Table 2. During the study period, the need for MV (78.4% vs 91.9%; *P* = 0.036) and vasopressors (75.0% vs 90.3%; *P* = 0.020) was significantly more frequent in the IPA group. No significant difference was found with regard to antibiotic, steroid and intravenous immunoglobulin therapy or to the proportion of patients with indwelling catheters between the IPA and non-IPA groups.

**Table 2:** **Clinical characteristics of critically ill immunocompromised patients**^**a**^

**Variables**	**All patients (*n*** **= 150)**	**No IPA (*n*** **= 88, 58.7%)**	**IPA (*n*** **= 62, 41.3%)**	**No IPA vs IPA *P *** **value**
Life-sustaining treatments,^b^*n* (%)				
Need for mechanical ventilation	126 (84.0)	69 (78.4)	57 (91.9)	0.036
Need for vasopressor	122 (81.3)	66 (75.0)	56 (90.3)	0.020
Need for RRT	38 (25.3)	24 (27.3)	14 (22.6)	0.571
Indwelling catheter,^c^*n* (%)				
Urinary catheter	146 (97.3)	85 (96.6)	61 (98.4)	0.643
CVC	141 (94.0)	81 (92.0)	60 (96.8)	0.308
Drug therapy,^d^*n* (%)				
Antifungal drugs	117 (78.0)	63 (71.6)	54 (87.1)	0.028
Corticosteroids^e^	105 (70.0)	57 (64.8)	48 (77.4)	0.107
IVIG^f^	43 (28.7)	20 (22.7)	23 (37.1)	0.067
Antibiotics for GNB^g^	147 (98.0)	86 (97.7)	61 (98.4)	>0.999
Antibiotics for GPB	98 (65.3)	56 (63.6)	42 (67.7)	0.728
Antiviral drugs	73 (48.7)	37 (42.0)	36 (58.1)	0.068
TMP-SMX	55 (36.7)	29 (33.0)	26 (41.9)	0.303
Outcomes				
ICU duration (days)	10.0 (11.0)	9.0 (10.0)	11.0 (14.0)	0.325
Hospital duration, (days)	29.0 (48.0)	30.0 (46.0)	27.0 (54.0)	0.681
ICU mortality, *n* (%)	64 (42.7)	29 (33.0)	35 (56.5)	0.005
Hospital mortality, *n* (%)	78 (52.0)	37 (42.0)	41 (66.1)	0.005
28-day mortality, *n* (%)	72 (48.0)	34 (38.6)	38 (61.3)	0.008
60-day mortality, *n* (%)	80 (53.3)	40 (45.5)	40 (64.5)	0.030
90-day mortality, *n* (%)	96 (64.0)	46 (52.3)	50 (80.6)	<0.001

^a^*CVC* central venous catheter, *GNB* Gram-negative bacilli, *GPB* Gram-positive bacteria, *IPA* invasive pulmonary aspergillosis, *IVIG* intravenous immunoglobulin, *RRT* renal replacement therapy, *TMP-SMX* trimethoprim/sulfamethoxazole. Continuous variables are expressed as the means ± SD or medians (IQR). All other data are raw numbers (%). ^b^Variable defined as life-sustaining treatment for 24 hours or longer during the study. ^c^Variable defined as the proportion of patients with catheter placed within the first 3 days following ICU admission. ^d^Variable defined as systemic drug therapy for 48 hours or longer during the ICU stay. ^e^Corticosteroid therapy was defined according to the 2008 EORTC-MSG criteria [6]: mean minimum dose of 0.3 mg/kg/day prednisone equivalent for more than 3 weeks during ICU stay. Other corticosteroids should be converted into equivalent dose of prednisone before calculation and comparison. ^f^Routine treatment was immunoglobulin 10 g/day. ^g^Variable defined as all GNB except multi-drug-resistant bacteria.

The ICU mortality rate (56.5% vs 33.0%; *P* = 0.005), hospital mortality rate (66.1% vs 42.0%; *P* = 0.005), 28-day mortality (61.3% vs 38.6%; *P* = 0.008), 60-day mortality (64.5% vs 45.5%; *P* = 0.03) and 90-day mortality (80.6% vs 52.3%; *P* < 0.001) were all significantly higher in the IPA group than in the non-IPA group. There was no difference found between the two groups regarding the median ICU duration and hospital duration.

The lungs were the most common source of infection, followed by the bloodstream (Table 3). Chest CT scans of 150 CIIC patients were defined as “positive,” “specific” or “negative” on the basis of the 2005 ATS guidelines and the 2008 EORTC-MSG consensus definitions by two independent senior radiologists, respectively, including 85 reviewed by one and 65 reviewed by the other. Compared with the actual number of IPA patients diagnosed on the basis of the 2008 EORTC-MSG consensus criteria (the standard method), the diagnostic efficiency of two radiologists was 0.71 (95% CI 0.68 to 0.73) and 0.69 (95% CI 0.66 to 0.73), with Cohen’s κ values of 0.339 and 0.338, respectively. Compared with the non-IPA group, higher proportions of patients had positive (85.5% vs 53.4%; *P* < 0.001) and specific (41.9% vs 10.2%; *P* < 0.001) chest CT findings in the IPA group. Important laboratory and microbiological parameters at the time of ICU admission, including Clinical Pulmonary Infection Score (CPIS) [20], procalcitonin (PCT) level, BG level, C-reactive protein (CRP) level and coexisting pathogens, did not differ between the two groups (Table 3).

**Table 3:** **Microbiological characteristics of critically ill immunocompromised patients**^**a**^

**Variables**	**All patients (*n*** **= 150)**	**No IPA (*n*** **= 88, 58.7%)**	**IPA (*n*** **= 62, 41.3%)**	**No IPA vs IPA *P *** **value**
Sites of infection, *n* (%)				
Lung	113 (75.3)	66 (75.0)	47 (75.8)	>0.999
Lung and BSI^b^	33 (22.0)	19 (21.6)	14 (22.6)	>0.999
Lung, BSI and intraabdominal	2 (1.3)	1 (1.1)	1 (1.6)	-
Lung, BSI and urogenital	1 (0.7)	1 (1.1)	0	-
Lung, BSI and soft tissue (skin)	1 (0.7)	1 (1.1)	0	-
Positive chest CT findings,^c^*n* (%)	100 (66.7)	47 (53.4)	53 (85.5)	<0.001
Radiologist 1,^d^*n* = 85 (100%)	55 (64.7)	27 (52.9)	28 (82.4)	0.006
Radiologist 2,^e^*n* = 65 (100%)	45 (69.2)	20 (54.1)	25 (89.3)	0.003
Specific chest CT findings,^f^*n* (%)	35 (23.3)	9 (10.2)	26 (41.9)	<0.001
Radiologist 1,^d^*n* = 85 (100%)	19 (22.4)	5 (9.8)	14 (41.2)	0.007
Radiologist 2,^e^*n* = 65 (100%)	16 (24.6)	4 (10.8)	12 (42.9)	0.001
CPIS score at ICU admission	8.3 (3.0)	8.2 (4.0)	8.6 (2.5)	0.161
Infection marker at ICU admission				
PCT level (ng/ml)	1.1 (4.2)	0.9 (4.1)	1.5 (4.4)	0.35
BG level (pg/ml)	5.0 (15.8)	5.0 (11.5)	5.0 (38.6)	0.26
CRP level (mg/L)	92.7 ± 73.5	90.9 ± 76.9	95.5 ± 68.4	0.718
Coexisting pathogens, *n* (%)				
CMV^g^	55 (36.7)	27 (30.7)	28 (45.2)	0.086
PCP	20 (13.3)	8 (9.1)	12 (19.4)	0.088
*Candida* species	60 (40.0)	40 (45.5)	20 (32.3)	0.128
MDR GNB^h^	99 (66.0)	55 (62.5)	44 (71.0)	0.299
GPB	43 (28.7)	21 (23.9)	22 (35.5)	0.144
GNB^i^	122 (81.3)	70 (79.5)	52 (83.9)	0.532

^a^*BG* (1,3)-β-d-glucan, *BSI* bloodstream infection, *CMV* cytomegalovirus, *CPIS* Clinical Pulmonary Infection Score, *CRP* C-reactive protein, *CT* computed tomography, *GNB* Gram-negative bacteria, *GPB* Gram-positive bacteria, *IPA* invasive pulmonary aspergillosis, *MDR* multi-drug-resistant, *PCPPneumocystis carinii* pneumonia, *PCT* procalcitonin. Continuous variables are expressed as the mean ± SD or the medians (IQR). All other data are raw numbers (%). ^b^Variable defined as recovery of any bacterium or fungus in one or more blood cultures taken in the presence of clinical evidence of infection following ICU admission. Patients in whom *Staphylococcus* (non-*S. aureus*) was isolated from blood cultures were not eligible, except if two or more consecutive samples grew the same species harboring the same antibiotic resistance pattern [10]. ^c^Variable defined as patients who developed a new and persistent radiographic infiltrate. ^d^The proportion of 85 critically ill immunocompromised (CIIC) patients with positive and specific chest CT findings in the IPA and non-IPA groups defined by radiologist 1. ^e^The proportions of 65 CIIC patients with positive and specific chest CT findings in the IPA and non-IPA groups defined by radiologist 2. ^f^Specific chest CT findings of IPA were defined according to the 2008 EORTC-MSG criteria [6]: a well-defined dense lesion (with or without a halo sign), a crescent sign and a cavity. ^g^Including patients with a positive CMV pp65 antigen, polymerase chain reaction or immunoglobulin M detection. ^h^Variable included MDR *Pseudomonas aeruginosa*, MDR *Acinetobacter baumannii* and MDR *Klebsiella pneumoniae*. ^i^Variable defined as all GNB except MDR GNB.

During the study period, we obtained 79 isolates of *Aspergillus* spp. from 62 CIIC patients. Of these isolates, 35 (44.3%) were *Aspergillus fumigates*, 18 (22.8%) were *Aspergillus flavus* and 11 (13.9%) were *Aspergillus niger*. Among the 62 CIIC patients, a single *Aspergillus* spp. isolate was found in 23 patients (37.1%) and two or more isolates were found in 10 patients (16.1%). *Candida* spp. isolates were present in 20 (32.3%) of the 62 patients, and other mold isolates were found in 25 patients (40.3%).

### Comparison of immune parameters by invasive pulmonary aspergillosis diagnosis

B-cell counts (D1: p = 0.048, D3: p = 0.043), CD3^+^ T-cell counts (D1: p = 0.018, D3: p = 0.001, and D10: p = 0.001, respectively), CD8^+^ T-cell counts (D1: p = 0.002, D3: p < 0.001, and D10: p < 0.001, respectively), CD28^+^CD4^+^ T-cell counts (D1: p = 0.028, D3: p = 0.049, and D10: p = 0.022, respectively), and CD28^+^CD8^+^ T-cell counts (D1: p = 0.003, D3: p < 0.001, and D10: p < 0.001, respectively) were significantly lower in the IPA than the non-IPA group. No significant differences were found in other immune parameters between the groups, including white blood cell (WBC) count, neutrophil granulocyte (NG) count, complement factors, and immunoglobulins (Table 4).

**Table 4:** **Analysis of immune parameters in critically ill immunocompromised patients**^**a**^

**Parameters**	**D1 (*n*** **= 150)**			**D3 (*n*** **= 130)**			**D10 (*n*** **= 104)**		
	**No IPA (*n*** **= 88)**	**IPA (*n*** **= 62)**	***P *** **value**	**No IPA (*n*** **= 79)**	**IPA (*n*** **= 51)**	***P *** **value**	**No IPA (*n*** **= 64)**	**IPA (*n*** **= 40)**	***P *** **value**
WBC (cells/mm^3^)	9,779.3 ± 6,754.6	8,896.0 ± 4,446.2	0.189	9,671.5 ± 5,314.7	9,459.8 ± 5,182.2	0.825	8,676.1 ± 4,217.4	9,261.7 ± 5,565.8	0.551
NG (cells/mm^3^)	8,554.8 ± 6,533.6	7,208.8 ± 4,205.0	0.162	8,498.6 ± 5,134.3	8,128.5 ± 4,929.7	0.688	7,237.7 ± 3,764.8	7,843.8 ± 5,463.3	0.513
NK (cells/mm^3^)	40.5 (72.3)	36.5 (59.3)	0.537	41.0 (52.0)	29.0 (57.0)	0.425	45.0 (80.8)	33.5 (50.5)	0.105
LB (cells/mm^3^)	64.5 (132.5)	43.0 (52.3)	0.048	86.0 (136.0)	51.0 (87.0)	0.043	71.0 (151.5)	50.5 (73.8)	0.311
CD3^+^ T (cells/mm^3^)	456.6 ± 299.0	342.5 ± 270.1	0.018	440.3 ± 217.2	250.0 (170.0)	0.001	531.0 ± 245.0	255.5 (342.5)	0.001
CD4^+^ T (cells/mm^3^)	206.4 ± 156.4	182.5 ± 175.3	0.120	185.1 ± 124.1	129.0 (135.0)	0.376	226.4 ± 143.9	121.5 (181.5)	0.095
CD28^+^CD4^+^ T (cells/mm^3^)	129.0 (174.0)	89.0 (152.0)	0.028	159.5 ± 107.5	91.0 (106.0)	0.049	189.0 ± 125.8	93.5 (179.3)	0.022
CD8^+^ T (cells/mm^3^)	232.1 ± 169.0	138.5 (110.8)	0.002	249.3 ± 157.0	100.0 (105.0)	<0.001	294.1 ± 163.3	99.0 (120.3)	<0.001
CD28^+^CD8^+^ T (cells/mm^3^)	104.7 ± 84.9	67.7 ± 61.9	0.003	115.2 ± 71.0	63.5 ± 59.6	<0.001	130.4 ± 77.6	59.0 (75.3)	<0.001
C3 (mg/dl)	78.4 (49.7)	71.9 ± 27.9	0.510	78.6 ± 33.1	77.6 ± 28.6	0.993	87.5 ± 51.1	79.7 ± 29.8	0.420
C4 (mg/dl)	17.1 ± 9.9	18.4 ± 9.6	0.463	16.7 ± 9.4	20.2 ± 12.0	0.104	17.5 (15.3)	18.9 ± 10.3	0.728
IgA (mg/dl)	167.7 ± 106.0	161.8 ± 106.1	0.758	170.4 ± 97.6	165.4 ± 102.9	0.802	168.4 ± 95.3	198.1 ± 121.4	0.204
IgG (mg/dl)	1,092.3 ± 641.9	977.8 ± 556.0	0.299	966.0 (576.0)	1,170.3 ± 620.5	0.556	1,123.3 ± 510.9	1,165.6 ± 597.9	0.724
IgM (mg/dl)	67.0 (64.0)	55.5 (56.5)	0.093	80.0 (62.5)	62.0 (53.0)	0.267	87.0 (83.3)	113.7 ± 113.1	0.904

^a^*C3* complement factor 3, *C4* complement factor 4, *D1* day 1, *D3* day 3, *D10* day 10, *Ig* immunoglobulin, *IPA* invasive pulmonary aspergillosis, *LB* B lymphocyte, *NG* neutrophil granulocyte, *NK* natural killer, *WBC* white blood cell. Continuous variables are expressed as means ± SD or medians (IQR).

To identify the prediction of IPA in CIIC patients, we applied multivariate logistic regression analysis for the parameters that demonstrated a *P* value less than 0.05 in the univariate analysis. With respect to comparison of pre-ICU variables, clinical and microbiological variables and immune parameters, 13 included in univariate logistic regression analysis showed significance for prediction of IPA in CIIC patients: older patients (*P* = 0.01), patients who needed MV (*P* = 0.016) or vasopressor infusion (*P* = 0.032), patients with positive or specific chest CT findings (*P* < 0.001, respectively), lower levels of CD3^+^ T-cell counts (D3: *P* = 0.042, D10: *P* = 0.003), CD8^+^ T-cell counts (D1: *P* = 0.007, D3: *P* < 0.001, and D10: *P* < 0.001, respectively) and CD28^+^CD8^+^ T-cell counts (D1: *P* = 0.007, D3: *P* < 0.001, and D10: *P* = 0.002, respectively).

Multivariate logistic regression analysis (Table 5) identified four independent predictors for the presence of an IPA diagnosis in CIIC patients: CD8^+^ T-cell counts (D3 and D10) (odds ratio (OR) 0.34, 95% confidence interval (CI) 0.23 to 0.46; OR 0.68; 95% CI 0.56 to 0.80), CD28^+^CD8^+^ T-cell counts (D3) (OR 0.73, 95% CI 0.61 to 0.86) and CD3^+^ T-cell counts (D10) (OR 0.81, 95% CI 0.63 to 0.98). Table 6 shows in detail the values of standard error, Wald’s coefficient, OR along with 95% CI, and the level of statistical significance. PCA was applied to adjust for the possible multicollinearity among immune parameters measured on D1, D3 and D10. According to the results of Kaiser-Meyer-Olkin (KMO) and Bartlett’s Test of Sphericity (KMO *P* < 0.5 and Bartlett’s Test of Sphericity *P* > 0.05), the immune parameters included in our regression model have no significant multicollinearity and will not cause overadjustment of IPA in CIIC patients.

**Table 5:** **Multivariate logistic regression analysis of factors predicting invasive pulmonary aspergillosis in critically ill immunocompromised patients**^**a**^

**Variables**	**β**	**SEM**	**Wald’s coefficient**	**OR (95% CI)**	***P *** **value**
CD3^+^ T (D10)	−0.217	0.134	2.612	0.805 (0.633 to 0.977)	0.023
CD8^+^ T (D3)	−1.074	0.356	9.095	0.342 (0.227 to 0.457)	0.003
CD8^+^ T (D10)	−0.384	0.166	5.358	0.681 (0.562 to 0.800)	0.009
CD28^+^CD8^+^ T (D3)	−0.312	0.147	4.501	0.732 (0.605 to 0.859)	0.018

^a^*D3* day 3, *D10* day 10, *CI* confidence interval, *OR* odds ratio, *SEM* standard error of the mean.

**Table 6:** **Comparison of immune parameters in critically ill immunocompromised patients with invasive pulmonary aspergillosis according to 28-day mortality**^**a**^

**Parameters**	**28-day mortality from D1 (*n*** **= 62)**			**28-day mortality from D3 (*n*** **= 51)**			**28-day mortality from D10 (*n*** **= 40)**		
	**Survivors (*n*** **= 24)**	**Nonsurvivors (*n*** **= 38)**	***P *** **value**	**Survivors (*n*** **= 24)**	**Nonsurvivors (*n*** **= 27)**	***P *** **value**	**Survivors (*n*** **= 24)**	**Nonsurvivors (*n*** **= 16)**	***P *** **value**
WBC (cells/mm^3^)	10,373.8 ± 6,566.5	7,733.2 ± 4,585.7	0.067	9,379.6 ± 5,066.3	9,154.8 ± 5,425.7	0.880	9,727.9 ± 6,185.9	9,440.0 ± 5,696.3	0.883
NG (cells/mm^3^)	8,855.5 ± 5,688.0	6,995.3 ± 4,337.1	0.157	7,885.4 ± 4,960.6	8,022.3 ± 5,247.7	0.923	7,770.1 ± 5,832.1	8,593.3 ± 5,788.2	0.663
LB (cells/mm^3^)	64.5 (132.5)	43.0 (52.3)	0.118	79.0 (107.5)	33.0 (44.0)	0.055	70.0 (89.0)	65.5 ± 89.6	0.147
NK (cells/mm^3^)	70.0 ± 62.0	48.8 ± 51.1	0.149	72.2 ± 62.3	29.0 ± 32.3	0.004	80.8 ± 73.6	36.4 ± 40.4	0.019
CD3^+^ T (cells/mm^3^)	547.5 ± 581.3	291.1 ± 230.1	0.049	487.5 ± 438.6	223.6 ± 199.6	0.011	472.9 ± 297.4	231.8 ± 226.9	0.006
CD4^+^ T (cells/mm^3^)	256.1 ± 274.1	178.6 ± 192.7	0.196	264.4 ± 287.9	146.4 ± 186.8	0.085	253.9 ± 209.3	154.3 ± 218.2	0.155
CD28^+^CD4^+^ T (cells/mm^3^)	211.7 ± 253.1	84.5 (139.0)	0.104	110.5 (151.5)	60.0 (122.0)	0.015	210.6 ± 198.9	69.0 (89.3)	0.040
CD8^+^ T (cells/mm^3^)	187.0 (164.5)	105.2 ± 70.4	<0.001	141.5 (115.8)	70.1 ± 41.3	<0.001	218.3 ± 187.1	66.6 ± 37.7	0.001
CD28^+^CD8^+^ T (cells/mm^3^)	95.0 (77.3)	42.9 ± 33.1	<0.001	106.6 ± 98.8	28.8 ± 20.0	0.001	107.2 ± 88.9	31.3 ± 18.6	<0.001
C3 (mg/dl)	66.6 ± 26.5	73.1 ± 27.3	0.393	80.5 ± 32.9	75.3 ± 25.1	0.554	80.5 ± 32.7	78.5 ± 26.2	0.846
C4 (mg/dl)	17.3 ± 9.1	18.4 ± 9.9	0.702	20.5 ± 10.5	19.9 ± 13.5	0.886	19.8 ± 11.4	17.7 ± 8.8	0.568
IgA (mg/dl)	195.9 ± 167.0	162.3 ± 110.3	0.387	190.0 ± 127.3	145.9 ± 76.0	0.166	227.4 ± 138.1	156.2 ± 79.5	0.067
IgG (mg/dl)	1,226.5 ± 651.4	881.9 ± 512.7	0.053	1,337.4 ± 707.4	1,038.0 ± 519.8	0.117	1,349.4 ± 673.7	903.1 ± 343.5	0.057
IgM (mg/dl)	86.5 ± 66.4	65.0 ± 49.6	0.222	92.4 ± 90.4	80.2 ± 69.0	0.618	101.8 ± 74.5	130.8 ± 154.3	0.470

^a^*C3* complement factor 3, *C4* complement factor 4, *D1* day 1, *D3* day 3, *D10* day 10, *Ig* immunoglobulin, *NG* neutrophil granulocyte, *LB* B lymphocyte, *NK* natural killer, *WBC* white blood cell. Continuous variables are expressed as means ± SD or medians (IQR).

### Comparison of immune parameters in survivors and nonsurvivors

The patients with IPA were divided into survivors and nonsurvivors according to 28-day mortality. Compared with the nonsurvivors, the following were significantly higher in survivors and exhibited a progressive increase from D1 to D10: NK cell counts (D3: *P* = 0.004, D10: *P* = 0.019), CD3^+^ T-cell counts (D1: *P* = 0.049, D3: *P* = 0.011 and D10: *P* = 0.006, respectively), CD28^+^CD4^+^ T-cell counts (D3: *P* = 0.015 and D10: *P* = 0.04), CD8^+^ T-cell counts (D1: *P* < 0.001, D3: *P* < 0.001 and D10: *P* = 0.001, respectively) and CD28^+^CD8^+^ T-cell counts (D1: *P* < 0.001, D3: *P* = 0.001 and D10: *P* < 0.001, respectively). Beyond that, no differences between survivors and nonsurvivors were found in other immune parameters studied (Table 6).

**Figure 2:**
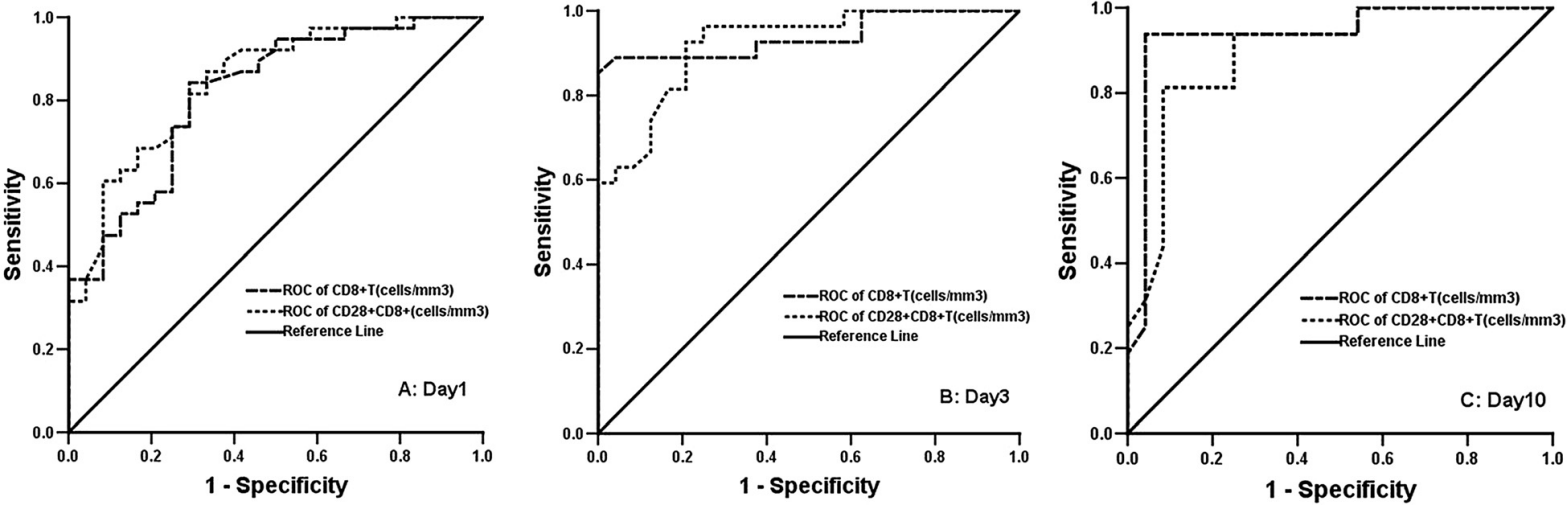
**Receiver operating characteristic (ROC) curve analysis of discriminatory ability of CD8**^**+ **^**and CD28**^**+**^**CD8**^**+ **^**T-cell counts to predict 28-day mortality in chronically ill immunocompromised patients with invasive pulmonary aspergillosis.** Area under the curve values on day 1 **(A)**, day 3 **(B)** and day 10 **(C)** following ICU admission.

**Figure 3:**
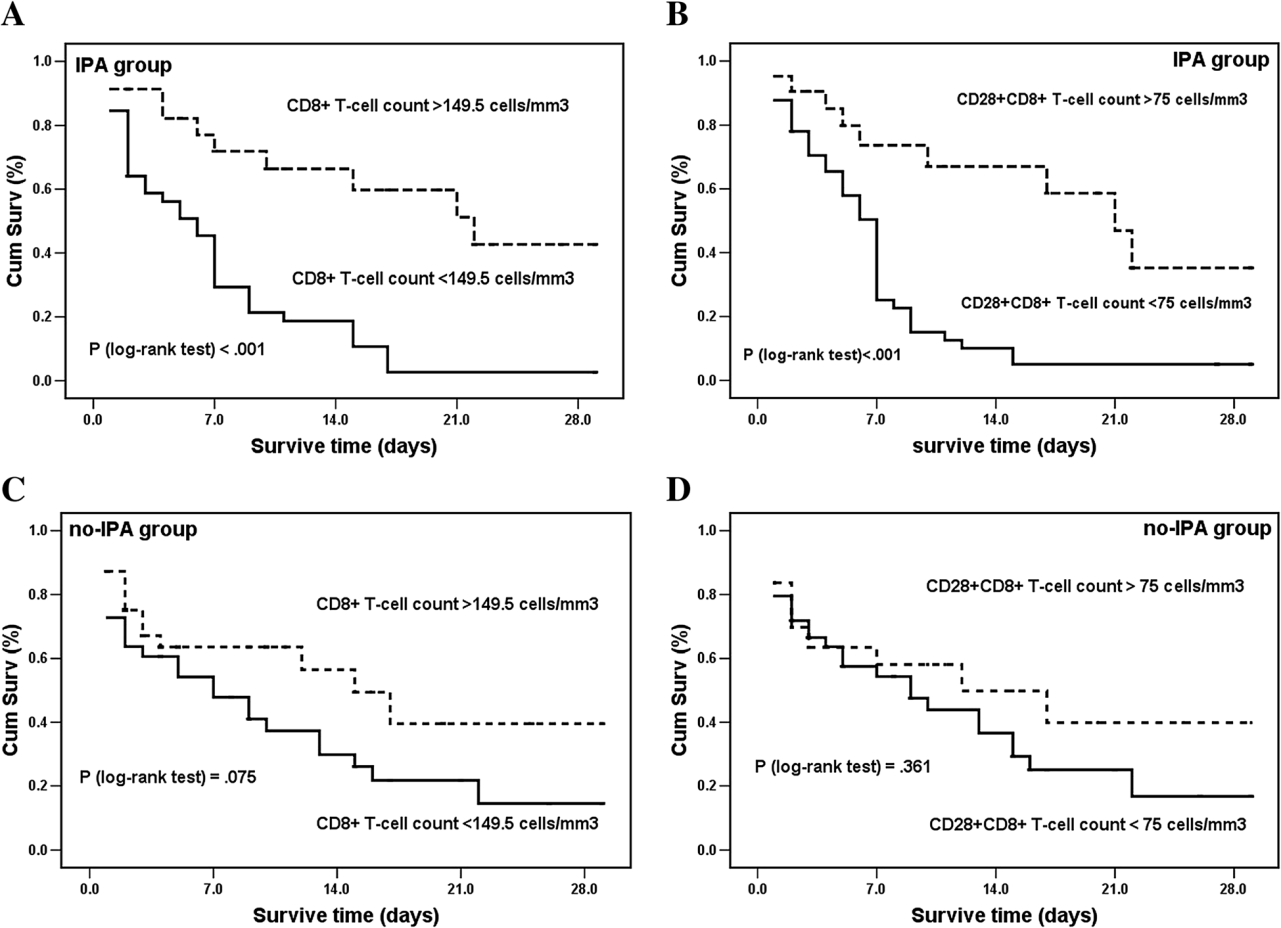
**Kaplan-Meier analysis of survival probabilities in chronically ill immunocompromised patients with and without invasive pulmonary aspergillosis.** Patients with invasive pulmonary aspergillosis (IPA) **(A)** and **(B)** and without IPA **(C)** and **(D)**. Survival measured according to CD8^+^ T-cell counts more (or less) than 149.5 cells/mm^3^ and CD28^+^CD8^+^ T-cell counts more (or less) than 75 cells/mm^3^ at the time of ICU admission. Survival time was censored on day 28. Cum Surv: cumulative survivors.

To assess the discrimination of prognosis, we applied ROC analysis with the immune parameters that differed significantly between survivors and nonsurvivors in CIIC patients with IPA according to 28-day mortality (Table 7). The CD8^+^ and CD28^+^CD8^+^ T-cell counts had greater discriminatory ability than the other immune parameters to predict 28-day mortality in CIIC patients with IPA, with area under the curve (AUC) values shown in Figure 2: CD8^+^ T-cell counts for D1 (AUC 0.82, 95% CI 0.71 to 0.92; *P* < 0.001), D3 (AUC 0.94, 95% CI 0.87 to 0.99; *P* < 0.001) and D10 (AUC 0.94, 95% CI 0.85 to 0.99; *P* < 0.001); and CD28^+^CD8^+^ T-cell counts for D1 (AUC 0.84, 95% CI 0.75 to 0.94; *P* < 0.001), D3 (AUC 0.92, 95% CI 0.85 to 0.99; *P* < 0.001) and D10 (AUC 0.90, 95% CI 0.79 to 0.99; *P* < 0.001). The cutoff values of CD8^+^ and CD28^+^CD8^+^ T-cell counts at ICU admission to predict 28-day mortality were 149.5 and 75 cells/mm^3^, with sensitivities of 84% and 87% and specificities of 71% and 67%, respectively. The optimal cutoff values of CD8^+^ and CD28^+^CD8^+^ T-cell counts on D10 had sensitivities of 94% and 81% and specificities of 96% and 92%, respectively, for predicting 28-day mortality (Table 8). Analysis of Kaplan-Meier survival curves provided evidence that a CD8^+^ T-cell count less than 149.5 cells/mm^3^ (logrank test; *P* < 0.001) and a CD28^+^CD8^+^ T-cell count <75 cells/mm^3^ (logrank test; *P* < 0.001) at ICU admission were associated with lower survival probabilities in CIIC patients with IPA (Figure 3A and 3B). On the contrary, the same cutoff values of CD8^+^ (logrank test; *P* = 0.075) and CD28^+^CD8^+^ T-cell counts (logrank test; *P* = 0.361) stated above had nothing to do with the survival probabilities in CIIC patients without IPA (Figure 3C and 3D).

**Table 7:** **Receiver operating characteristic curve analysis of immune parameters predicting 28-day mortality in critically ill immunocompromised patients with invasive pulmonary aspergillosis**^**a**^

**Parameters**^**b **^**(cells/mm**^**3**^**)**	**28-day mortality from D1 (*n*** **= 62)**			**28-day mortality from D3 (*n*** **= 51)**			**28-day mortality from D10 (*n*** **= 40)**		
	**Cutoff value (cells/mm**^**3**^**)**	**AUC (95% CI)**	***P *** **value**	**Cutoff value (cells/mm**^**3**^**)**	**AUC (95% CI)**	***P *** **value**	**Cutoff value (cells/mm**^**3**^**)**	**AUC (95% CI)**	***P *** **value**
NK		–		41.0	0.78 (0.66 to 0.91)	0.001	23.0	0.73 (0.57 to 0.90)	0.013
CD3^+^ T	215.0	0.68 (0.55 to 0.82)	0.017	235.5	0.77 (0.64 to 0.90)	0.001	243.0	0.75 (0.59 to 0.91)	0.007
CD28^+^CD4^+^ T		–		61.5	0.70 (0.55 to 0.85)	0.015	108.5	0.69 (0.52 to 0.87)	0.040
CD8^+^ T	149.5	0.82 (0.71 to 0.92)	<0.001	96.5	0.94 (0.87 to 0.99)	<0.001	90.5	0.94 (0.85 to 0.99)	<0.001
CD28^+^CD8^+^ T	75.0	0.84 (0.75 to 0.94)	<0.001	56.5	0.92 (0.85 to 0.99)	<0.001	46.0	0.90 (0.79 to 0.99)	<0.001

^a^*AUC* area under the curve, *CI* confidence interval, *D1* day 1, *D3* day 3, *D10* day 10, *NK* natural killer. ^b^Variable included the immune parameters that differed significantly between survivors and nonsurvivors in critically ill immunocompromised patients with invasive pulmonary aspergillosis according to 28-day mortality (Table 6).

**Table 8:** **CD8**^**+ **^**and CD28**^**+**^**CD8**^**+ **^**T-cell counts predicting 28-day mortality in critically ill immunocompromised patients with invasive pulmonary aspergillosis**^**a**^

**Parameters (cells/mm**^**3**^**)**	**Time**	**Cutoff value (cells/mm**^**3**^**)**	**Sensitivity (95% CI)**	**Specificity (95% CI)**	**Positive predictive value (95% CI)**	**Negative predictive value (95% CI)**	**PA**
CD8^+^ T	D1	149.5	0.84 (0.73 to 0.96)	0.71 (0.53 to 0.89)	0.82 (0.71 to 0.94)	0.74 (0.56 to 0.92)	0.79
	D3	96.5	0.89 (0.77 to 0.99)	0.96 (0.88 to 0.99)	0.96 (0.88 to 0.99)	0.89 (0.76 to 0.99)	0.92
	D10	90.5	0.94 (0.82 to 0.99)	0.96 (0.88 to 0.99)	0.94 (0.82 to 0.99)	0.96 (0.88 to 0.99)	0.95
CD28^+^CD8^+^ T	D1	75.0	0.87 (0.76 to 0.98)	0.67 (0.48 to 0.86)	0.81 (0.68 to 0.93)	0.76 (0.58 to 0.94)	0.79
	D3	56.5	0.93 (0.83 to 0.99)	0.79 (0.63 to 0.95)	0.83 (0.70 to 0.97)	0.91 (0.78 to 0.99)	0.86
	D10	46.0	0.81 (0.62 to 0.99)	0.92 (0.81 to 0.99)	0.87 (0.70 to 0.99)	0.88 (0.75 to 0.99)	0.88

^a^*D1* day 1, *D3* day 3, *D10* day 10, *CI* confidence interval, *PA* Percentage of agreement.

## Discussion

To the best of our knowledge, this is the first cohort study to evaluate the quantitative changes in host immune status in CIIC patients with pulmonary infection and the potential role of these changes in the generation and prognosis of IPA. Our results confirm the recent improvements in understanding the potential role of host immunity in the development of opportunistic infection and provide evidence that assessment of the systemic levels of several key immune parameters in CIIC patients could be valuable for early diagnosis and outcome prediction in patients with IPA.

Over the past two decades, IPA has been known as an important cause of morbidity and mortality only in patients with compromised host defenses. During that time, people have realized that the susceptibility of critically ill patients is due to increased incidence of IPA. Unlike patients who are simply immunocompromised, the immune responses of CIIC patients are closely associated with and greatly influenced by the infectious etiologies of critical illness [21]. Therefore, the immune status of CIIC patients with IPA is dictated by the interaction between pathogen and host and should have prognostic value in this condition. In the present study, we monitored levels of lymphocyte subpopulations (T, B and NK cells), complement factors C3 and C4 and immunoglobulins IgA, IgG and IgM in 150 CIIC patients with or without IPA at three times during the ICU stay and showed that differences in the systemic levels of key immune parameters could influence their clinical outcome. We believe that our measurement of these parameters as the first comprehensive assessment of the immune status in CIIC patients with pulmonary infection is the first attempt to predict risk and outcome of IPA from the point of view of immune susceptibility.

In the present study, we found that CD8^+^ and CD28^+^CD8^+^ T-cell counts were significantly lower in both survivors and nonsurvivors among CIIC patients with or without IPA, and they were independent predictors for higher risk of IPA. This result is in accord with the outcome analyses depending on the 28-day mortality and provides new evidence supporting the critical roles of CD8^+^ T cells in the immune responses to fungal infection. As we know, fungal infections are associated with impaired cell-mediated immunity [22]. Previous studies have shown that CD4^+^ T-cell responses are critical for protection from invasive fungal infections (IFIs), whereas CD8^+^ T cells have not been examined. Recent studies have focused on discovery and validation of immune mechanisms against IFIs, which have demonstrated the protective roles of CD8^+^ T cells during fungal infections, including production of the signature cytokine interferon γ (IFN- γ) [23], release of the antimicrobial peptides [24] and lysis of fungus-containing phagocytes [25]. In addition, CD8^+^ T cells can protect mice from *Cryptococcus neoformans* infection in the absence of CD4^+^ T cells [26], and the expansion of cytotoxic, class I-restricted, *A. fumigatus*-specific CD8^+^ T-cell clones from human peripheral blood has also suggested that CD8^+^ T cells contribute to cell-mediated defense [27]. The CD28 molecule is known to be the most important second signal receptor for T lymphocytes. CD28 costimulation has been implicated in a wide array of T-cell responses, including T-cell proliferation, antigen-stimulated differentiation, activation and cytokine production [28]. The expression levels of CD28 molecules on the surface of T lymphocytes have been reported to be greatly decreased in severely infected patients [29], and reduced expression of CD28 is thought to be an individual risk factor for the increased mortality of infected patients. In addition, the requirement for CD28 costimulation has been found to differ between CD4^+^ and CD8^+^ T-cell populations and appears to depend on antigen presentation [30]. Our study confirmed this observation by identifying CD28 costimulation in CD8^+^ cells, but not in CD4^+^ T cells, as independent predictors for higher risk and early mortality in patients with IPA. Although the interaction between significantly reduced CD8^+^ and CD28^+^CD8^+^ T-cell counts and the emergence and progression of IPA in CIIC patients remains unclear, this close correlation provides new clues for the early diagnosis of IPA in CIIC patients.

The role of NK cells in fungal infections is unclear and controversial. In our study, patients with IPA showed lower levels of NK and CD8^+^ T cells following ICU admission. Lower levels of NK cells were associated with increased 28-day mortality rates in CIIC patients with IPA. In accordance with our findings, recent advances in NK cell biology have revealed that NK cells exert sophisticated biological functions, participating with antigen-presenting and T cells in the cellular response against pathogens [31], and perforin has recently been reported to be a major mediator of NK cell activity against *A. fumigatus* hyphae [32]. These results highlight the protective effect of NK cells in their interactions with *A. fumigatus*, prompting further investigations concerning the antifungal activity of NK cells. The specific biological activities of the complement system that contribute to host resistance are multifaceted and interdependent [33]. There were no differences in the kinetic patterns of complement factors or immunoglobulins between survivors and nonsurvivors among CIIC patients with IPA, which is consistent with previous studies. A better understanding of the diverse biological activities and their governing mechanisms will be indispensable to determining the predictive potential of this cell variable.

Despite significant advances in the prevention, diagnosis and treatment of infection in CIIC patients, it remains a major cause of morbidity, increased hospital length of stay and increased total costs. The significantly higher ICU mortality rates among these patients are caused in part by the higher incidence of infection. Although there is a rapidly increasing evidence base in intensive care medicine, CIIC patients have been excluded in most interventional trials for the management of severe sepsis (activated protein C, adjunctive corticosteroids, goal-directed resuscitation), acute lung injury and failure of other organs [34-36]. Most previous clinical studies of CIIC patients with IPA have been retrospective analyses [6] with the common limits of low case numbers, long time periods and inevitably missing data, which are expected, given the patient population and the specificity of the disease itself. A total of 150 critically ill patients with appropriate immunocompromised host factors and documented pulmonary infection were prospectively and consecutively included in our study. MV and vasopressors were required in more than 90% of the patients, which is higher than in previous studies. No patient was transferred to the ICU for postoperative monitoring. All these characteristics are fully representative of the clinical presentation of CIIC patients with IPA. Unlike other studies, however, 112 (74.7%) of our cases had an underlying disease of the immune system and 101 (65.3%) were treated with glucocorticoids, which may have been prescribed because they are the first-choice drug for the treatment of immune system diseases. In the present study, we found that the dose of steroids and the proportions of positive and specific chest CT findings were significantly higher in patients with IPA than in those without it. As suggested previously [37], the sensitivity and specificity of chest CT images in the diagnosis of IPA have been variable and seemingly affected by a multitude of factors, including evolution of clinical practices and changes in the high-risk patient populations. Animal models have shown that different forms of immunosuppressant-induced IPA have completely different histopathological features [38]. Diffuse and extensive consolidation and inflammation in glucocorticoid-induced IPA may partially explain the higher value of chest CT images in diagnosis of IPA in the present study.

Some limitations of our study must be acknowledged. First, owing to the influence of traditional cultures and the potentially devastating complications, autopsy and tissue biopsy were greatly limited in this study, leading to the absence of proven IPA and the subsequent influence on the evaluation. However, this study does to some extent reflect the actual clinical settings in the vast majority of developing countries, and a practicable solution is needed to resolve the problem. Second, two serological markers, GM index and BG, were not routinely monitored in this study, a factor which needs to be improved in future research. Third, TBAs are commonly used for the diagnosis of pulmonary infection because of the limited availability of BAL or PSB in our hospital, which might put the accuracy of IPA diagnosis into question. Actually, in the immunocompromised host, except in some special groups of patients (for example, after lung transplantation), cultures of *Aspergillus* in respiratory secretions are usually indicative of invasive disease with positive predictive value as high as 80% to 90% [39]. Moreover, direct microscopic examination of sputum could be helpful to improve the sensitivity of cultures in the diagnosis of IPA [40].

## Conclusion

The immune status of CIIC patients with IPA is dictated by the interaction between pathogen and host could be valuable for clinical diagnosis and outcome prediction. Our investigation suggests that CD8^+^ and CD28^+^CD8^+^ T-cell counts are significantly lower in CIIC patients with IPA than in non-IPA patients and are independent predictors for higher risk of IPA. Lower CD8^+^ and CD28^+^CD8^+^ T-cell counts in CIIC patients with IPA are associated with early mortality in IPA and may be valuable for outcome prediction.

## Key messages

● Identifying quantitative changes of key cellular and humoral parameters in CIIC patients could be valuable for early diagnosis and outcome prediction in cases of IPA.

● CD8^+^ and CD28^+^CD8^+^ T-cell counts were significantly lower in CIIC patients with IPA than in non-IPA patients and were independent predictors for higher risk of IPA.

● Lower CD8^+^ and CD28^+^CD8^+^ T-cell counts in CIIC patients with IPA were associated with early mortality in IPA and may be valuable for outcome prediction.

● The predictive potential of CD8^+^ and CD28^+^CD8^+^ T-cell counts in CIIC patients with IPA should prompt further investigations concerning the antifungal activities and the governing mechanisms of the cell variables.

## Abbreviations

APACHE II: Acute Physiology and Chronic Health Evaluation II; ATS: American Thoracic Society; AUC: Area under the curve; BAL: Bronchoalveolar lavage; BG: (1,3)-β-D-glucan; BSI: Bloodstream infection; CIIC: Critically ill immunocompromised; CMV: Cytomegalovirus; COPD: Chronic obstructive pulmonary disease; CPIS: Clinical Pulmonary Infection Score; CT: Computed tomography; CRP: C-reactive protein; CVC: Central venous catheter; C3: Complement factor 3; C4: Complement factor 4; EORTC-MSG: European Organization of the Research and Treatment of Cancer/Mycoses Study Group; GCP: good clinical practice; GM: Galactomannan; GNB: Gram-negative bacilli; GPB: Gram-positive bacteria; HAP: Hospital-acquired pneumonia; HLOS: Hospital length of stay; IFI: Invasive fungal infection; IFN-γ: Interferon γ; Ig: Immunoglobulin; IVIG: Intravenous immunoglobulin; IPA: Invasive pulmonary aspergillosis; KMO: Kaiser-Meyer-Olkin; LB: B lymphocyte; MCTD: Mixed connective tissue disease; MDR: Multi-drug-resistant; MPA: Microscopic polyangitis; MV: Mechanical ventilation; NK: Natural killer; PA: Percentage of agreement; PBMC: Peripheral blood mononuclear cell; PCA: Principal component analysis; PCP: *Pneumocystis carinii* pneumonia; PCT: Procalcitonin; PSB: Protected specimen brush; PMNL: Polymorphonuclear leukocyte; PUMCH: Peking Union Medical College Hospital; ROC: Receiver operating characteristic; SLE: Systemic lupus erythematosus; SOFA: Sequential Organ Failure Assessment; TBA: Tracheobronchial aspirate; TMP-SMX: Trimethoprim-sulfamethoxazole.

## Competing interests

The authors declare that they have no competing interests.

## Authors’ contributions

NC and HW are joint authors and contributed equally to this manuscript. DL, YL, NC and HW contributed to the design of the study and drafted the manuscript. NC and HW obtained the data. DL, NC and HW participated in data analysis and interpretation of the results. All authors read and approved the final manuscript for publication.
